# Matching in the House of Surgery: A Retrospective Analysis of Trends Across Surgical Subspecialties From 2015 to 2024

**DOI:** 10.7759/cureus.102708

**Published:** 2026-01-31

**Authors:** Mandy Hsu, Dennis J Head, Abdul-Jawad J Majeed, Jay D Raman

**Affiliations:** 1 Urology, Penn State College of Medicine, Hershey, USA; 2 Urology, Penn State Health Milton S. Hershey Medical Center, Hershey, USA

**Keywords:** match, medical education, residency, surgery, workforce

## Abstract

Background: Residency positions in surgical subspecialties are highly competitive due to numerous applicants and limited positions. This study analyzes a decade of trends in applicant numbers and match rates across surgical subspecialties.

Methods: Publicly available data from 2015 to 2024 were collected from the American Urologic Association and National Resident Matching Program for Neurologic Surgery, Otolaryngology, Orthopedic Surgery, Plastic Surgery, Urology, and Vascular Surgery. Descriptive statistics, linear regression, and analysis of variance were conducted.

Results: Of 29,483 registered applicants, 19,535 (66.3%) matched. Otolaryngology had the highest match rate (74.5%), while Vascular Surgery had the lowest (52.7%). Registered and matched applicants varied significantly by year within each specialty. For instance, Orthopedic Surgery demonstrated particularly significant growth in both registered applicants and matched applicants. Generalized linear models also revealed significant year-specialty interactions, suggesting inconsistent trends across specialties.

Conclusions: Matching into surgical specialties has grown more difficult given increased applicant numbers. Though matched applicants increased, growth is inconsistent and does not meet demand. Addressing this gap may require changes such as application process reforms or better resource distribution.

## Introduction

In the United States, residency spots for surgical subspecialties are highly competitive, with large numbers of applicants looking for a limited number of open positions each year. For most surgical subspecialties, this process is done through the National Resident Matching Program (NRMP), a non-profit independent organization that uses a mathematical algorithm to pair medical graduates with residency positions based on ranked preferences [1]. For Urology, this process is conducted through the American Urological Association (AUA) and the Society of Academic Urology (SAU) with their own matching algorithm [2].

The demand for surgical subspecialty residency spots has only continued to grow. Indicators of rising demand include the increasing number of applicants, the number of matched applicants, and match rates, which have shown that interest has often outpaced the number of available positions for almost all surgical subspecialties. In 2024, the NRMP reported fill rates across surgical specialties to be greater than 99%, exceeding the average fill rate of 93.8% across all specialties combined [3]. Most recently, in 2024, across Neurological Surgery, Plastic Surgery, Orthopedic Surgery, Otolaryngology, and Vascular Surgery, only three out of the available 1,852 positions went unfilled [4]. This is despite the fact that many surgical residency programs have expanded the number of positions available and continue to plan for more. Therefore, though essentially all surgical residency positions are being filled, it is unclear that this rise is sufficient to keep up with demand from applicants.

Matching into a surgical subspecialty is how medical graduates receive the training necessary to become practicing surgeons. However, given the rapidly aging population of the United States, more surgeons are approaching retirement age, with fewer surgeons entering the workforce to replace them and care for patients. Over a fourth of surgeons are older than 65 years old, and for Orthopedic Surgery, Plastic Surgery, and Urology, this percentage is even greater [3]. It is projected that if the current workforce of surgeons retires between the ages of 63 and 67, there will be a shortage of about 10,000-19,900 surgeons by the year 2036 [3]. This shortage is also compounded by the effects of an unequal distribution of physicians, especially in more rural areas of the United States. Given these upcoming needs, it is critical to understand trends of the match process for surgical subspecialties, which will undoubtedly have implications for how the surgical workforce will change in size and distribution in the future.

The aim of this study is to examine trends in the number of registered applicants, matched applicants, and match rates across various surgical subspecialties from 2015 to 2024, using data from the NRMP and AUA. By characterizing these trends, this study will offer insight into the evolving landscape of surgical residency recruitment and training. These findings will inform future medical graduates who are applying to surgical specialties, guide program planning for surgical residencies, and contribute to the broader discussion surrounding the future of the surgical workforce.

## Materials and methods

Yearly, the NRMP (nrmp.org) and AUA (auanet.org) publish data on their websites regarding the number of registered applicants, the number of matched applicants, and match rates. The data for surgical subspecialties including Neurologic Surgery, Orthopedic Surgery, Otolaryngology, Plastic Surgery, and Vascular Surgery for the years 2015-2024 was collected from the NRMP. This data was collected as published by the NMRP, without exclusion. Data regarding the specialties Thoracic Surgery, General Surgery, and Obstetrics and Gynecology were excluded due to either low numbers, not being a surgical subspecialty, or having overlap between surgery and primary care, respectively. The data for the Urology match for the years 2015-2024 was collected from the AUA. This data was also collected as published without exclusion. Very few positions go unfilled in these surgical subspecialties, so the number of matched applicants was also considered as a proxy for the number of available residency positions.

The data was transferred to a master Excel file (Microsoft Corporation, Redmond, Washington, United States) containing the information from the NMRP and AUA. General descriptives, frequencies, linear regression, and one-way analysis of variance (ANOVA) with post hoc Tukey's at a significance level of 0.05 were used to analyze the data with IBM SPSS Statistics for Windows, Version 30.0 (IBM Corp., Armonk, New York, United States). No study approval number was needed for this study as the information is retrospective, de-identified, and already publicly available by the NMRP and AUA.

## Results

**Figure 1:**
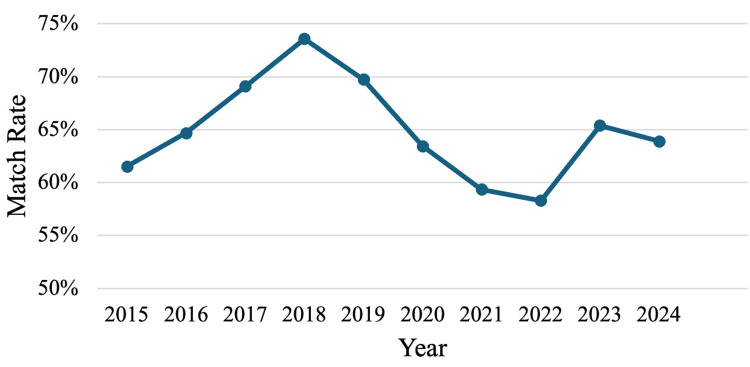
Line graph of average match rates for all surgical subspecialties combined between 2015 and 2024

A total of 29,483 applicants registered to match into a surgical subspecialty between 2015 and 2024, out of which 19,535 applicants successfully matched into a surgical subspecialty. This is an average match rate of 66.3% across all surgical subspecialty applicants during the past decade, with a range of 58.8-73.6% (Figure 1). Applicant numbers peaked in the year 2022, reaching 3,555 registered applicants. This was also the year of the lowest average match rate (58.3%). Between 2015 and 2024, there was a net increase of 826 registered applicants (+31%). The number of matched applicants increased from 1,703 in 2015 to 2,234 by 2024 (+31.2%). See Appendix 1 for applicant numbers and match rates. There was no statistically significant difference in average match rates across years.

**Figure 2:**
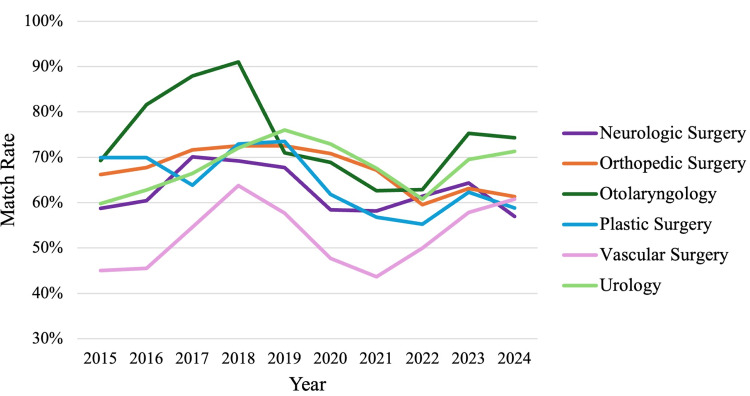
Match rates across surgical subspecialties during the time period of 2015-2024

A one-way ANOVA showed significant differences in match rates across surgical subspecialties (F(5,54)=11.782; p<0.001). Otolaryngology had the highest average match rate (74.5%), while Vascular Surgery had the lowest average match rate (52.7%). Neurologic Surgery, Orthopedic Surgery, Plastic Surgery, and Urology had match rates of 62.6%, 67.3%, 64.5%, and 67.9%, respectively. Overall, Vascular Surgery demonstrated a significantly lower match rate than all other specialties (p<0.05 for all comparisons). Otolaryngology had a significantly higher match rate than the specialties Neurologic Surgery (p=0.003), Plastic Surgery (p=0.018), and Vascular Surgery (p<0.001). With the exception of these findings, the average match rate of surgical specialties was not significantly different (Figure 2). See Appendix 2 for complete pairwise comparisons.

Utilizing a univariate general linear model (GLM), there was a significant effect of year on the number of applicants (F(1,48)=85.36; p<0.001), as well as significant differences in the number of registered applicants across surgical subspecialties (F(5,48)=11.52; p<0.001). The interaction between year and specialty was also significant, suggesting that the rate of change of registered applicants varied between specialties (Table 1). Orthopedic Surgery demonstrated a significant increase in applicants over time compared to other surgical subspecialties, with an average increase of 58.8 applicants per year (p<0.001) between 2015 and 2024. Separate linear regression showed that applicant numbers increased significantly within Neurologic Surgery, Otolaryngology, Plastic Surgery, Urology, and Vascular Surgery with average rates of 8.7, 21.5, 18.8, 11.8, and 7.4 applicants per year, respectively. These within-specialty trends were statistically significant on their own (p range: 0.033 to <0.001; Table 2). 

**Table 1:** Generalized linear model results for the registered applicants

Variable	F	df	P-value
Specialty	11.52	(5,48)	<0.001
Year	85.36	(1,48)	<0.001
Specialty×year	11.73	(5,48)	<0.001

**Table 2:** Linear regression results for the number of registered applicants by surgical subspecialty

Specialty	Slope (applicants/year)	P-value
Neurologic Surgery	8.73	0.016
Orthopedic Surgery	58.76	<0.001
Otolaryngology	21.48	0.015
Plastic Surgery	18.78	<0.001
Urology	11.82	0.033
Vascular Surgery	7.47	0.014

Another univariate GLM demonstrated a significant effect of year (F(1,48)=526.91; p<0.001) and specialty (F(5,48)=50.30; p<0.001) on the number of matched applicants. The interaction between year and specialty was also significant in this model, suggesting that the rate of change in matched applicants varied between surgical specialties (Table 3). Orthopedic Surgery demonstrated the largest increase in matched applicants over the past decade compared to other surgical subspecialties, with an average increase of 26.7 matched applicants per year (p<0.001). Otolaryngology (+10.6; p<0.001), Urology (+11.06; p<0.001), and Vascular Surgery (+5.1; p<0.001) also showed significant changes in the average number of matched applicants per year compared to other surgical subspecialties. Though not significantly different from other surgical subspecialties, Neurologic Surgery increased at an average rate of 3.74 matched applicants per year, and Plastic Surgery increased by an average of 7.67 matched applicants per year. Separate linear regression by specialty revealed that the number of matched applicants within each specialty increased significantly over time, with consistent upward trends across the past decade (p<0.001 for all; Table 4). 

**Table 3:** Generalized linear model results for the matched applicants

Variable	F	df	P-value
Specialty	50.30	(5,48)	<0.001
Year	526.91	(1,48)	<0.001
Specialty×year	51.80	5,48)	<0.001

**Table 4:** Linear regression results for the number of matched applicants by surgical specialty

Specialty	Slope (applicants/year)	P-value
Neurologic Surgery	3.74	<0.001
Orthopedic Surgery	26.69	<0.001
Otolaryngology	10.55	<0.001
Plastic Surgery	7.67	<0.001
Urology	11.06	<0.001
Vascular Surgery	5.12	<0.001

## Discussion

Our findings highlight notable disparities within match rates and applicant trends among surgical subspecialties over the past decade. The average match rate among surgical subspecialties has varied greatly, with a notable low of 58.3% in the year 2022. This occurred despite a peak in applicant numbers that year, perhaps demonstrating increased competition or a mismatch between the number of applicants and the number of matched applicants, which could be considered the number of available residency positions. Match rates rose back closer towards the average match rate of 66.3% in subsequent years, yet this rise was not consistent, highlighting the fluctuating competitiveness of surgical subspecialties.

Otolaryngology demonstrated the highest average match rate, significantly greater than other surgical subspecialties, including Neurologic Surgery, Plastic Surgery, and Vascular Surgery. In particular, Vascular Surgery had the lowest average match rate and was significantly lower than all other surgical subspecialties. This is similar to other Vascular Surgery match rate research that has demonstrated a ratio of nine applicants hoping to fill four positions annually within the subspecialty [5]. Within Otolaryngology, some work has explored the impact of the Program-Specific Paragraph (PSP) and Otolaryngology Resident Talent Assessment (ORTA), which were put in place in 2015 and 2016. The PSP made it so that applicants had to submit an extra paragraph to express interest in specific programs, while the ORTA provided telephone-based structured interviews to assess qualities of applicants not otherwise encompassed by their residency application. The PSP was made optional in 2018, while the ORTA became a post-match, rather than pre-match requirement starting in 2019. De Ravin et al. found that applicant numbers dropped while match rates rose during the implementation of these programs, and after the PSP and ORTA were made optional and post-match, there was an associated rise in applicant numbers, and match rates dropped once more [6]. The implementation of extra steps such as these, though often tedious for applicants to wade through, may contribute to the higher match rates seen in Otolaryngology. It could be worthwhile to further explore other factors that make Otolaryngology report significantly greater match rates compared to other surgical subspecialties to improve match rates. With this in mind, it is still important to note that the match rate of Otolaryngology, though highest in this study, is still below the average match rate across all specialties.

Separate linear regression analyses demonstrated that the number of applicants and matched applicants increased significantly over time within each surgical subspecialty, reflecting consistent growth in interest and training capacity across the past decade. This suggests that interest in surgical subspecialties remains strong and continues to grow yearly, while residency programs are also steadily opening more residency positions in an attempt to meet this demand.

Even so, the significant interaction between year and specialty in both GLMs demonstrates that the growth in applicant numbers and match rates is not uniform across surgical subspecialties. Orthopedic Surgery showed substantial increases in both the number of applicants and matched applicants, which may reflect larger residency class sizes and high workforce demand. To a significant but not as great degree, Otolaryngology, Urology, and Vascular Surgery also matched a significantly greater number of applicants. Despite this, the rise in matched applicants does not seem to align with the desire, for instance, in Orthopedic Surgery, where for the almost 60 additional applicants who were interested each year, only about 26 more matches were made. The discrepancy underscores the current constraints of training capacity and increased competition that do not meet the expanding interest in surgical subspecialties. Importantly, this issue is systemic, as unmatched applicants are often highly qualified individuals who have excelled both academically and clinically, but are unable to secure a position due to insufficient capacity rather than inadequate merit [7].

The slower rate of increase when it comes to the number of matched applicants in Neurologic Surgery and Plastic Surgery may warrant further investigation to determine whether factors such as program limitations, applicant self-selection, or other barriers are contributing to slower growth compared to other surgical subspecialties. These findings are especially concerning given that the Neurologic Surgery workforce is projected to have a shortage of about 11% by the year 2035, while Plastic Surgery may be facing a shortage of 25% by that time [8]. These shortages are particularly alarming considering growing concerns of upcoming surgeon burnout, an aging surgical workforce, and their downstream effects on patient access and outcomes [9,10]. One thought may be that our existing resources are not being distributed in the most economic manner. For instance, though the number of General Surgery positions has increased to 1,717 in 2024, which is a 12% increase since 2020, the specialty is predicted to have 104% adequacy by 2035 [4,8]. Yet, while these projections suggest adequacy in General Surgery, it is critical to note that aggregate numbers can mask severe rural shortages. Therefore, the most strategic allocation must also take this into consideration instead of solely reducing positions for other specialties. Other recent surgical match trends data have highlighted a growth in surgical available and filled positions, though this growth is mostly led by General Surgery [11]. Such imbalances suggest that surgical training positions, as well as the resources required for them, could be more strategically allocated across specialties to better meet long-term workforce needs and minimize projected future shortages. Furthermore, as noted by the American College of Surgeons, though legislation such as the Resident Physician Shortage Reduction Act of 2021 have been introduced to expand residency position numbers, such bills have been introduced by Congress numerous times in the past without any progress [12].

From the perspective of a residency program, limitations to expansion may include Accreditation Council for Graduate Medical Education (ACGME) requirements that mandate low faculty-to-resident ratios [13,14], as well as minimal availability of operative volume, faculty, and hospital resources to support more trainees. While applicant motivation is not measured or reported on in this observational study, previous literature has suggested that desired compensation may be one factor that contributes to high numbers of registered applicants. For instance, many medical students graduate with high amounts of debt, putting them in undesirable financial situations especially compared to peers of similar age already in the workforce. In 2019, almost three out of four graduating medical students graduated with debt, at a median of $200,000 [15]. This may factor into Bernstein's suggestion that the higher the mean salary of the specialty, the greater the number of applicants per available position. The top three specialties illustrated in this article happened to be three surgical subspecialties: Orthopedic Surgery, Plastic Surgery, and Otolaryngology [16]. If this were to be one of the main drivers propelling applicant interest in surgical subspecialties, addressing workforce shortages may require more than just increasing the number of training slots. Systemic targeted policy interventions, such as loan repayment incentives or adjustments to compensation structures [15], would be necessary to resolve the gap between applicant interest and matched positions.

There are a few limitations to this study. Not all surgical subspecialties are represented, as some require fellowships and are not represented by NRMP data, and others, such as Thoracic Surgery, have too few data points to analyze alongside other surgical specialties. Future studies can focus specifically on those specialties to provide insight into their match processes and the implications on possible future workforce shortages. Additionally, though rare, utilizing matched applicants as a proxy for available positions may slightly underestimate true capacity because there have historically been a few unmatched positions in these surgical subspecialties in specific years. Finally, this study identifies trends regarding applicants and match numbers utilizing publicly available aggregate data. Therefore, these findings do not reflect individual-level factors, such as academic scores and geographic preferences, that can influence match outcomes. Systemic changes, such as preference signaling, Step 1 becoming pass/fail, and even the impact of COVID-19, may have impacted applicant behaviors and outcomes in ways that may not be captured by this analysis.

## Conclusions

Matching into surgical subspecialties has only become more difficult over time, as the number of applicants expressing interest in surgical subspecialties is steadily growing. Even though the number of matched applicants, and, by proxy, available positions, has also increased over time, the growth in matched applicants is not consistent across surgical specialties and still does not meet applicant demands. To better address the growing gap between applicant demand and available residency positions, a variety of changes could be considered. Areas to look into include altering the residency application process for surgical subspecialties and optimizing the distribution of training resources to address the widening gap between supply and demand.

## Appendices

Appendix 1

**Table 5:** Number of registered applicants, matched applicants, and match rates by surgical subspecialty and year

Specialty	Year	% matched	Applicants matched	Applicants registered
Neurologic Surgery	2015	58.76%	208	354
Orthopedic Surgery	2015	66.20%	703	1062
Otolaryngology	2015	69.30%	298	430
Plastic Surgery	2015	69.90%	144	206
Vascular Surgery	2015	45.08%	55	122
Urology	2015	59.84%	295	493
Neurologic Surgery	2016	60.45%	214	354
Orthopedic Surgery	2016	67.77%	717	1058
Otolaryngology	2016	81.62%	302	370
Plastic Surgery	2016	69.91%	151	216
Vascular Surgery	2016	45.53%	56	123
Urology	2016	62.82%	294	468
Neurologic Surgery	2017	70.10%	218	311
Orthopedic Surgery	2017	71.67%	726	1013
Otolaryngology	2017	87.92%	291	331
Plastic Surgery	2017	63.82%	157	246
Vascular Surgery	2017	54.63%	59	108
Urology	2017	66.46%	317	477
Neurologic Surgery	2018	69.23%	225	325
Orthopedic Surgery	2018	72.57%	738	1017
Otolaryngology	2018	90.99%	303	333
Plastic Surgery	2018	72.93%	167	229
Vascular Surgery	2018	63.74%	58	91
Urology	2018	72.02%	314	436
Neurologic Surgery	2019	67.74%	231	341
Orthopedic Surgery	2019	72.52%	752	1037
Otolaryngology	2019	71.00%	328	462
Plastic Surgery	2019	73.50%	172	234
Vascular Surgery	2019	57.66%	64	111
Urology	2019	76.04%	330	434
Neurologic Surgery	2020	58.44%	232	397
Orthopedic Surgery	2020	70.81%	844	1192
Otolaryngology	2020	68.91%	348	505
Plastic Surgery	2020	61.86%	180	291
Vascular Surgery	2020	47.71%	73	153
Urology	2020	72.93%	353	484
Neurologic Surgery	2021	58.21%	234	402
Orthopedic Surgery	2021	67.18%	866	1289
Otolaryngology	2021	62.61%	350	559
Plastic Surgery	2021	56.84%	187	329
Vascular Surgery	2021	43.65%	79	181
Urology	2021	67.61%	357	528
Neurologic Surgery	2022	61.38%	240	391
Orthopedic Surgery	2022	59.52%	875	1470
Otolaryngology	2022	62.89%	361	574
Plastic Surgery	2022	55.27%	194	351
Vascular Surgery	2022	50.00%	84	168
Urology	2022	60.73%	365	601
Neurologic Surgery	2023	64.34%	240	373
Orthopedic Surgery	2023	63.09%	899	1425
Otolaryngology	2023	75.25%	371	493
Plastic Surgery	2023	62.35%	207	332
Vascular Surgery	2023	57.86%	92	159
Urology	2023	69.51%	383	551
Neurologic Surgery	2024	56.97%	241	423
Orthopedic Surgery	2024	61.33%	915	1492
Otolaryngology	2024	74.27%	381	513
Plastic Surgery	2024	58.84%	213	362
Vascular Surgery	2024	60.74%	99	163
Urology	2024	71.30%	385	540

Appendix 2

**Table 6:** Tukey's post hoc pairwise comparisons of match rates across surgical specialties

Specialty	Comparison specialty	P-value
Neurologic Surgery	Orthopedic Surgery	0.617
	Otolaryngology	0.003
	Plastic Surgery	0.986
	Urology	0.475
	Vascular Surgery	0.019
Orthopedic Surgery	Neurologic Surgery	0.617
	Otolaryngology	0.168
	Plastic Surgery	0.940
	Urology	1.000
	Vascular Surgery	<0.001
Otolaryngology	Neurologic Surgery	0.003
	Orthopedic Surgery	0.168
	Plastic Surgery	0.018
	Urology	0.256
	Vascular Surgery	<0.001
Plastic Surgery	Neurologic Surgery	0.986
	Orthopedic Surgery	0.940
	Otolaryngology	0.018
	Urology	0.862
	Vascular Surgery	0.003
Urology	Neurologic Surgery	0.475
	Orthopedic Surgery	1.000
	Otolaryngology	0.256
	Plastic Surgery	0.862
	Vascular Surgery	<0.001
Vascular Surgery	Neurologic Surgery	0.019
	Orthopedic Surgery	<0.001
	Otolaryngology	<0.001
	Plastic Surgery	0.003
	Urology	<0.001
